# Inframalleolar thrice distal puncture in a single endovascular treatment session for successful revascularization

**DOI:** 10.1186/s42155-023-00369-8

**Published:** 2023-03-29

**Authors:** Issei Ota, Tetsuya Nomura, Kenshi Ono, Yu Sakaue, Keisuke Shoji, Naotoshi Wada

**Affiliations:** Department of Cardiovascular Medicine, Kyoto Chubu Medical Center, 25, Yagi-Ueno, Yagi-cho, Nantan City, Kyoto 629-0197 Japan

**Keywords:** Critical limb-threatening ischemia, Infrapopliteal arterial disease, Endovascular treatment, Inframalleolar, Distal puncture

## Abstract

**Background:**

Most patients with chronic limb-threatening ischemia (CLTI) have infrapopliteal arterial disease, which are often challenging to treat. In endovascular treatment (EVT) for these complex lesions, establishing retrograde access is an essential option not only for guidewire crossing but also for device delivery. However, no EVT case has yet been reported requiring inframalleolar thrice distal puncture in a single EVT session so far.

**Case presentation:**

A 60-year-old CLTI patient with grade 3 Wound, Ischemia and foot Infection (WIfI) classification underwent EVT for occluded dorsal artery and posterior tibial artery. First, we conducted successful balloon angioplasty of the posterior tibial artery by establishing a retrograde approach via the lateral plantar artery. To treat the occlusion of the dorsal artery, we punctured the first dorsal metatarsal artery, and retrogradely advanced a guidewire to the dorsal artery occlusion; however, the microcatheter could not follow the guidewire. Therefore, we punctured the occluded distal anterior tibial artery and introduced the retrograde guidewire into the puncture needle. After guidewire externalization, we pulled up the retrograde microcatheter into the occlusion of dorsal artery using the “balloon deployment using forcible manner” technique. Thereafter, we were able to advance the antegrade guidewire into the retrograde microcatheter. After guidewire externalization, an antegrade balloon catheter was delivered and inflated for the purpose of dorsal artery dilation and hemostasis at the “needle rendezvous” point. Consecutively, balloon dilation was performed for puncture site hemostasis of the first dorsal metatarsal artery and complete hemostasis was achieved. Finally, we confirmed good vascular patency and favorable blood flow. After revascularization, transmetatarsal amputation was performed and the wound healed favorably.

**Conclusions:**

We can markedly increase the success rate of revascularization by effectively utilizing the retrograde approach in EVT for complex chronic total occlusions in infrapopliteal arterial diseases.

## Introduction

Chronic limb-threatening ischemia (CLTI) is a consequence of the progression of peripheral arterial disease, and has been widely known as a critical pathological entity associated with increased risk of cardiovascular death, myocardial infarction, or ischemic stroke. Most patients with CLTI have infrapopliteal (IP) arterial diseases, which are often challenging to treat (Sato et al. 2022). Development of endovascular treatment (EVT) techniques and devices have facilitated higher rates of successful revascularization. Particularly, establishing retrograde access after failure of the antegrade approach is a useful option not only for guidewire crossing but also for device delivery.

## Case presentation

**Fig. 1:**
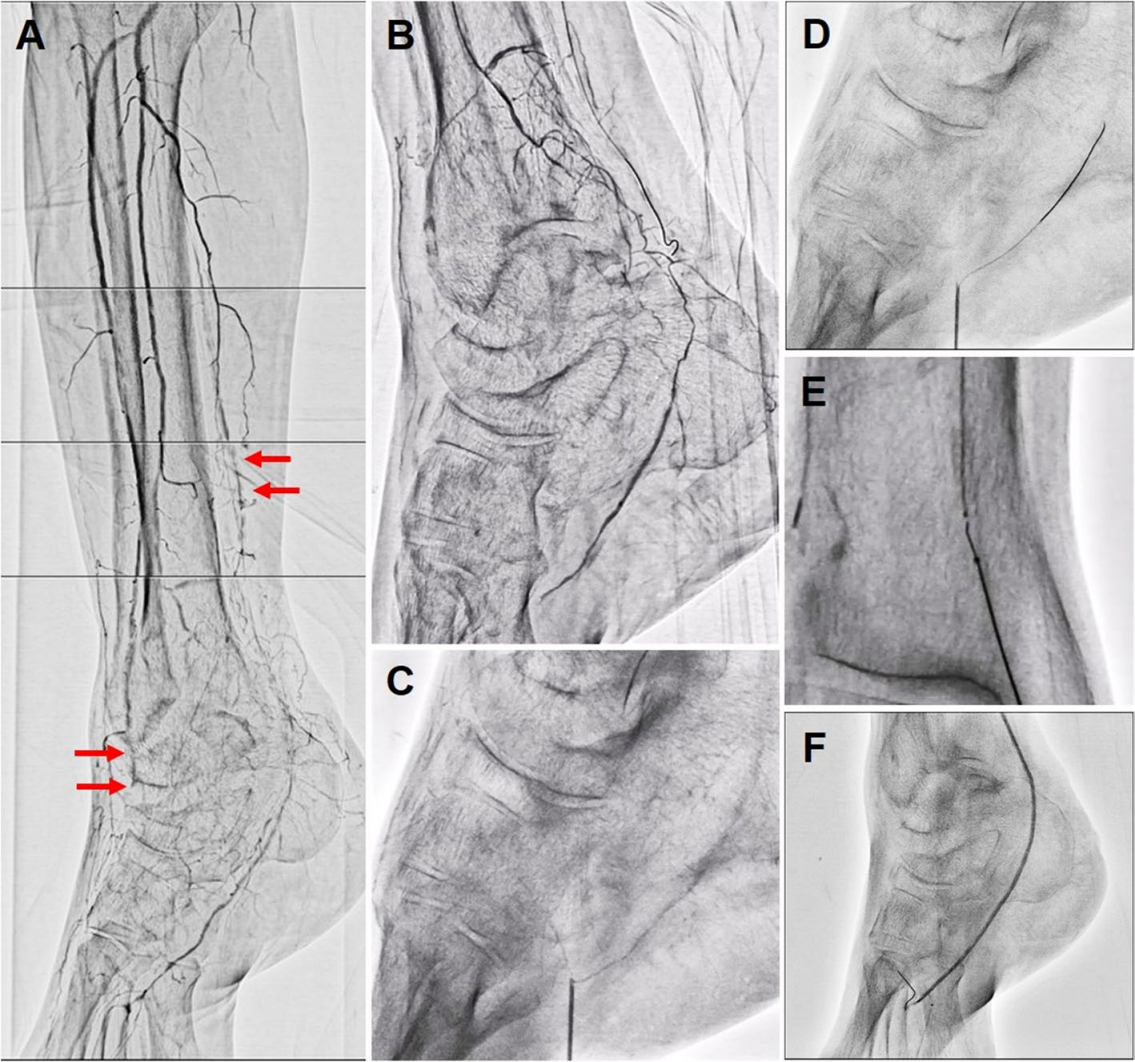
**a** Initial angiography showing occlusions of the distal anterior tibial artery (ATA) and mid posterior tibial artery (PTA) with severe calcification (Arrows). **b** The lateral plantar artery was perfused by collateral blood flow from the peroneal artery. **c** First distal puncture of the lateral plantar artery with angiographical guidance. **d** Gladius MG guidewire advancement into the lateral plantar artery. **e** Retrograde guidewire insertion into the antegrade microcatheter. **f** Successful balloon angioplasty of the occluded PTA.

A 60-year-old man who had been undergoing intermittent chronic hemodialysis for 35 years developed severe gangrene in all the digits of his right foot (Rutherford 5). Skin perfusion pressure (SPP) demonstrated 23 and 3 mmHg at dorsal and bottom of the foot, respectively. The Wound, Ischemia and foot Infection (WIfI) classification was stage 3 with W-1, I-3, fI-1. Medical treatment was not effective for the severe ischemic gangrene of this patient. Since non-invasive imaging findings revealed severe IP arterial diseases, we performed angiography. The patient received 100 mg aspirin and 75 mg clopidogrel prior to catheterization. We opted for antegrade puncture of the right common femoral artery and inserted a 4-Fr 10-cm long regular sheath (Terumo, Tokyo, Japan) into the right superficial femoral artery. Immediately after sheath insertion, an intra-arterial bolus of 5,000 units of unfractionated heparin was administered, and the activated clotting time was controlled to ≥ 250 s during the procedure. Angiography of the right lower extremity revealed occlusion of the distal anterior tibial artery (ATA) and mid posterior tibial artery (PTA) with severe calcification (Fig. 1a). The Global Limb Anatomic Staging System (GLASS) classification was stage III with femoropopliteal grade 0 and IP grade 4; inframalleolar (IM) disease with GLASS IM P2 was also present. Since optimal great saphenous vein (GSV) for bypass grafting was not available in this patient, we implemented EVT for limb salvage.

We exchanged the 4-Fr regular sheath for a 4.5-Fr Parent Plus 45 guide sheath (Medikit, Tokyo, Japan). The initial antegrade guidewire for the occluded PTA advanced into the subintimal space due to severe calcification, and we could not intentionally manipulate the guidewire anymore. At that point, as the lateral plantar artery was perfused by collateral blood flow from the peroneal artery (Fig. 1b), we established a retrograde approach by angiographically puncturing the lateral plantar artery with a 20-gauge puncture needle (Medikit, Tokyo, Japan) (Fig. 1c) and introduced the retrograde guidewire (Gladius MG: ASAHI INTECC, Aichi, Japan) into the antegrade microcatheter (Fig. 1d, e). After first guidewire externalization, we conducted successful balloon (Coyote 2.0-mm/150-mm: Boston Scientific, MA, USA) angioplasty of the PTA for 10 min, simultaneously completing hemostasis of the puncture point by balloon tamponade (Fig. 1f).

**Fig. 2:**
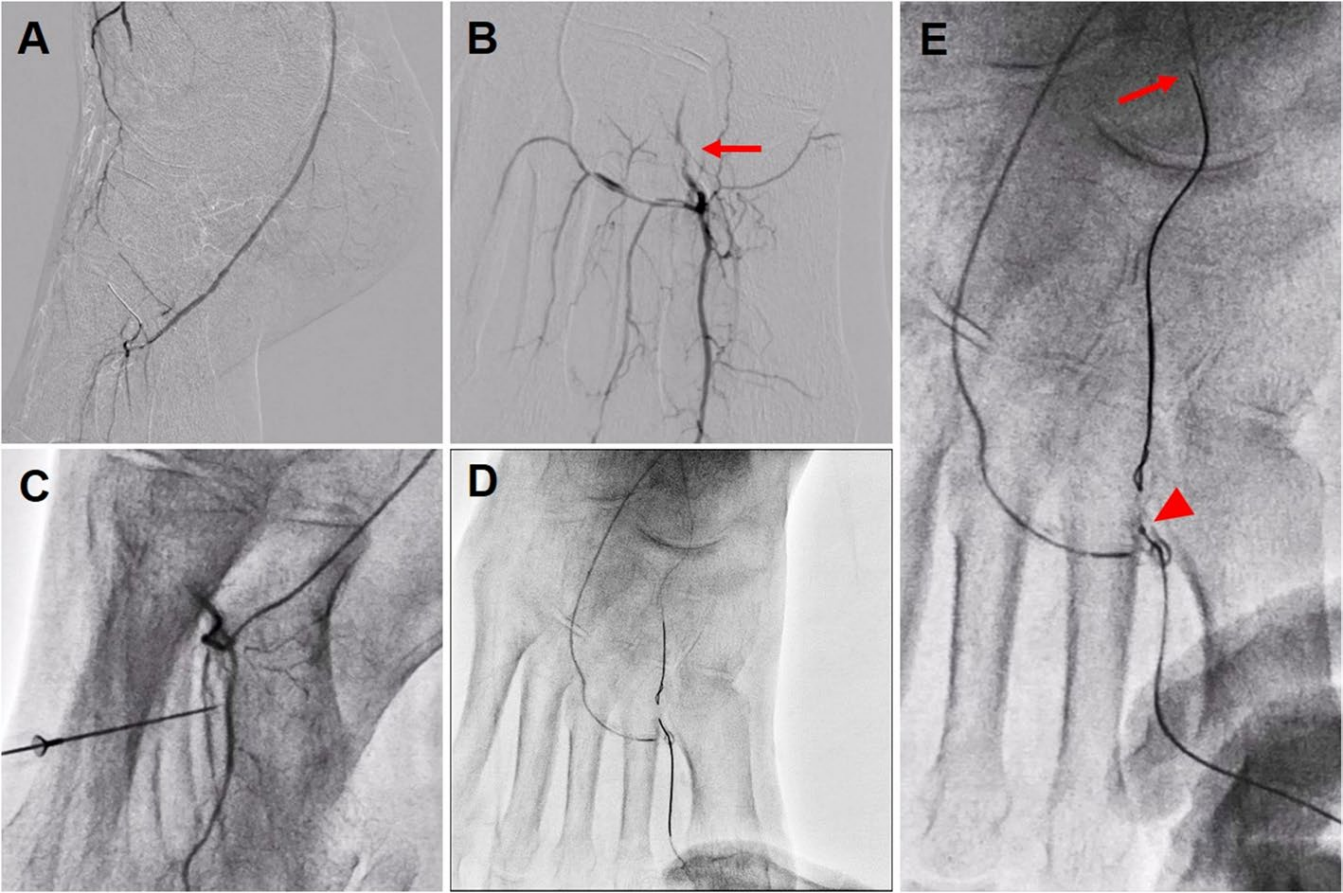
**a**, **b** Visualization of the pedal arch by microcatheter tip injection. Arrow indicating the distal end of the dorsal artery (DA) occlusion. **c** Second distal puncture of the first dorsal metatarsal artery for retrograde access. **d** Gladius MG guidewire penetration into the occluded DA. **e** The retrograde Naveed Hard30 guidewire advancement as far as distal ATA (Arrow). However, Corsair Armet microcatheter could not follow the guidewire (Arrowhead)

Next, since the antegrade guidewire via ATA could not pass through the occluded dorsal artery (DA), we tried to establish trans-pedal arch approach from the revascularized PTA. Microcatheter tip injection of contrast medium enabled clear visualization of distal end of the occluded DA (Fig. 2a,b). However, acute bend of the pedal arch hampered the guidewire penetration retrogradely into the DA occlusion. Therefore, we retrogradely punctured the first dorsal metatarsal artery under the angiographical guidance with 22-gauge indwelling needle (Surf flow: Terumo, Tokyo, Japan) (Fig. 2c) and advanced a guidewire (Gladius MG: ASAHI INTECC, Aichi, Japan) to the occluded DA (Fig. 2d). The retrograde guidewire (Naveed Hard30: Terumo, Tokyo, Japan) could advance as far as distal ATA along with the course of antegrade guidewire, but the microcatheter (Corsair Armet: ASAHI INTECC, Aichi, Japan) could not follow the guidewire (Fig. 2e).

**Fig. 3:**
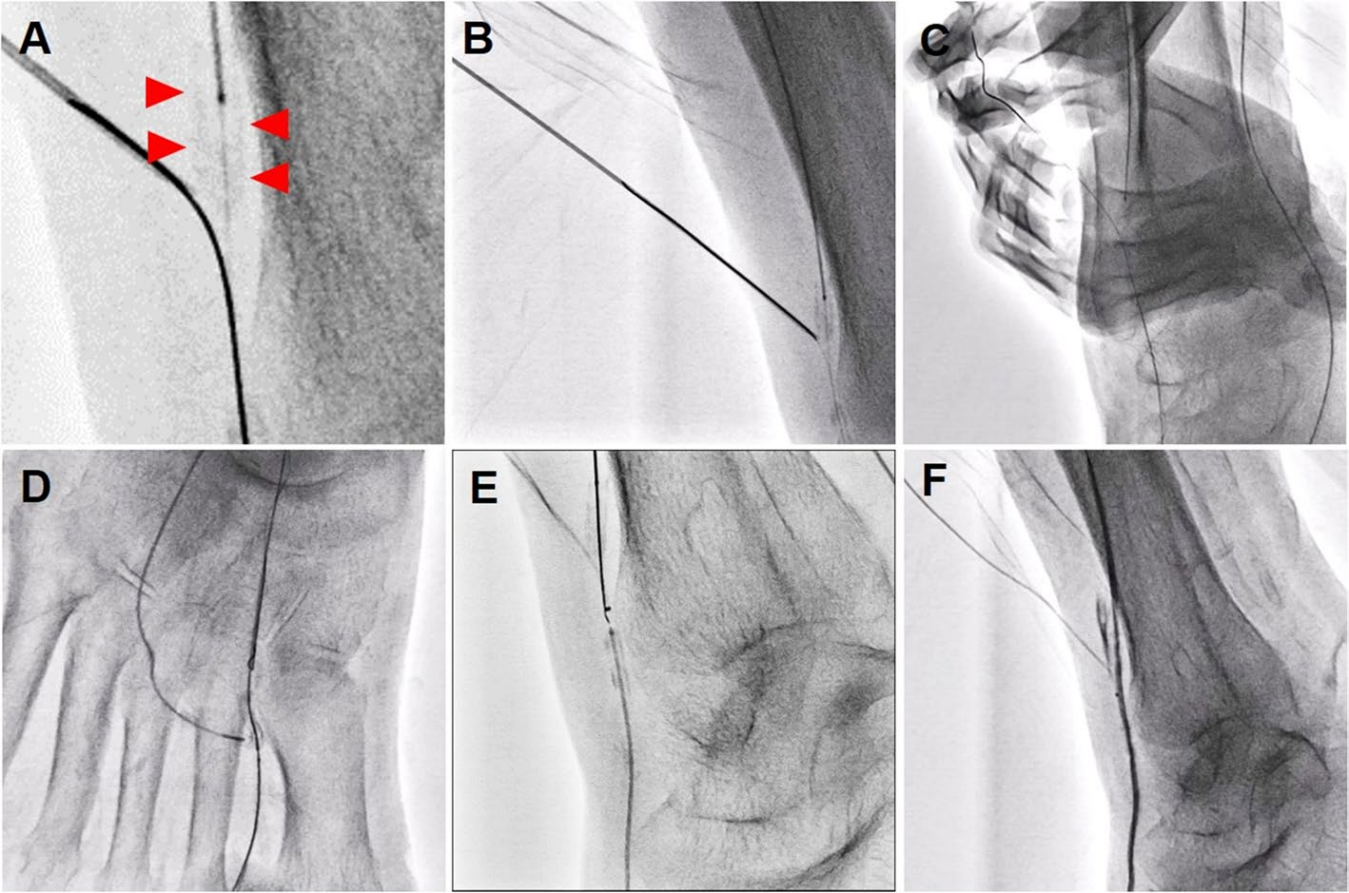
**a** Third distal puncture of the distal ATA occlusion making the vessel calcification as a target under fluoroscopy guidance (Arrowheads). **b** The retrograde Naveed Hard30 guidewire insertion into the puncture needle. **c** Second guidewire externalization. **d** Successful retrograde Corsair Armet microcatheter advancement into the occluded DA using the balloon deployment using forcible manner technique. **e** Antegrade AstatoXS 9–40 guidewire insertion into the retrograde microcatheter. **f** Prolonged balloon inflation for DA vessel dilation and hemostasis of the distal ATA puncture site

To make pull-through formation using Rendezvous technique in occluded DA, retrograde microcatheter advancement into the DA occlusion was mandatory. For that purpose, we punctured the occluded distal ATA with 20-gauge puncture needle (Medikit, Tokyo, Japan) making the vessel calcification as a target under fluoroscopy guidance (Fig. 3a) and introduced the retrograde Naveed Hard30 guidewire into the puncture needle (Fig. 3b). After the second time guidewire externalization (Fig. 3c), the puncture needle was exchanged for Ichibanyari PAD microcatheter (KANEKA: Osaka, Japan) and we pulled up the retrograde Corsair Armet microcatheter into the occluded DA using the BAlloon Deployment using FORcible Manner (BADFORM) technique (Nakabayashi et al. 2017) (Fig. 3d). Thereafter, we were able to advance the antegrade guidewire (AstatoXS 9–40: ASAHI INTECC, Aichi, Japan) into the retrograde microcatheter (Fig. 3e). After the third guidewire externalization, a Coyote antegrade balloon catheter was delivered into the occluded DA using the BADFORM technique and was inflated to dilate the vessel and for hemostasis at the distal ATA puncture site for approximately 10 min (Fig. 3f).

**Fig. 4:**
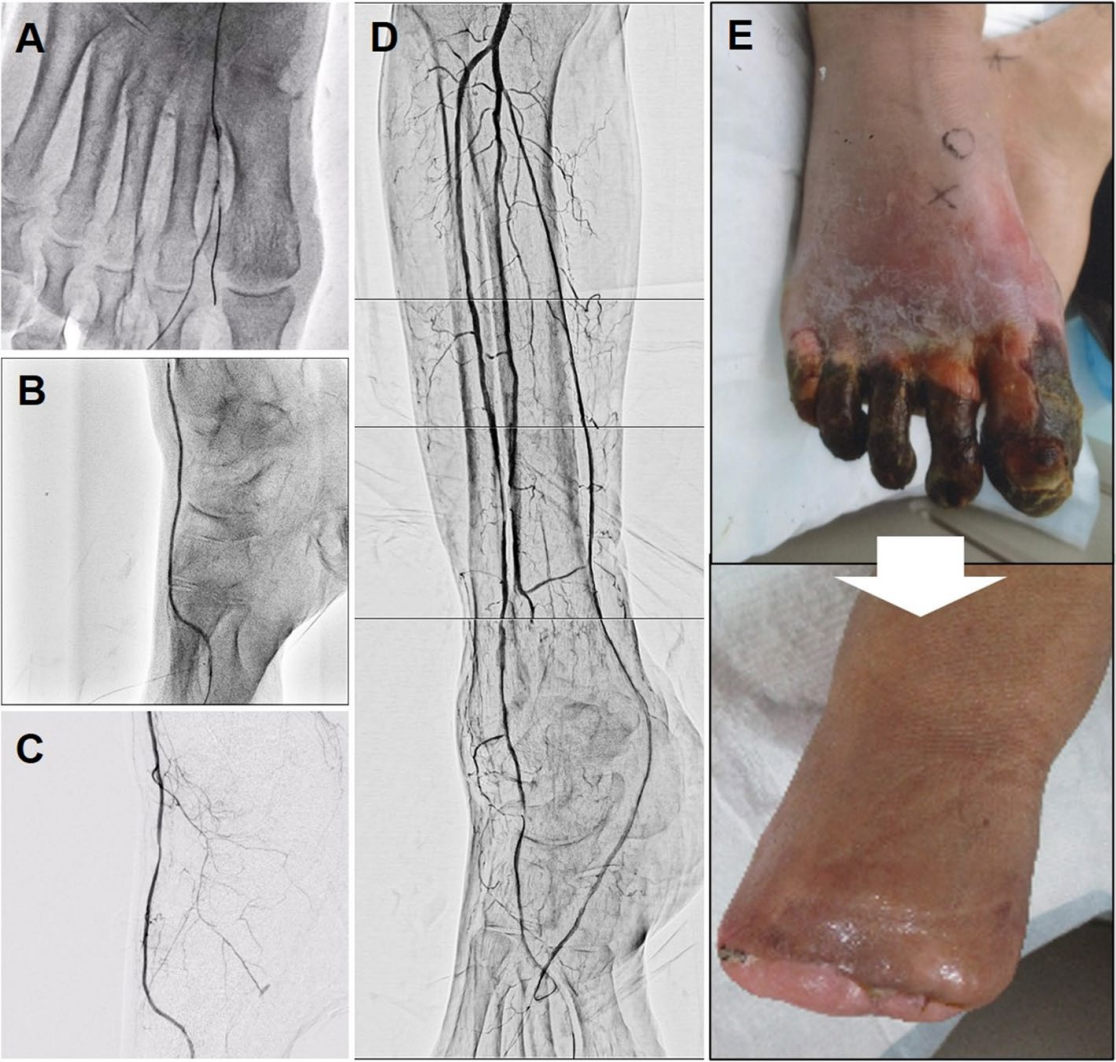
**a** Antegrade guidewire advancement to the first digital artery. **b** Balloon dilation to hemostat the puncture site of the first dorsal metatarsal artery. **c** Confirmation of complete hemostasis. **d** Final angiography showing good vascular patency and favorable blood flow. **e** (Upper panel) Severe gangrene in all the digits of the right foot before endovascular treatment. (Lower panel) Favorable wound healing after transmetatarsal amputation

Subsequently, antegrade guidewire was advanced to the first digital artery after releasing guidewire externalization (Fig. 4a). Subsequently, Coyote balloon dilation for about 5 min was added for puncture site hemostasis of the first dorsal metatarsal artery (Fig. 4b) and complete hemostasis was achieved (Fig. 4c). Finally, we confirmed good vascular patency and favorable blood flow (Fig. 4d). SPP restored to 47 and 39 mmHg at dorsal and bottom of the foot, respectively. Two weeks after revascularization, transmetatarsal amputation (TMA) was performed and the wound finally healed favorably (Fig. 4e).

## Discussion

The Global Vascular Guidelines (GVGs) recommend the preferred method of initial revascularization for CLTI to be based on assessment of anatomical complexity (GLASS stage) and limb severity (WIfI stage) (Conte et al. 2019). The IM anatomic modifier is also defined in the GVGs, but this modifier is not relevant to the GLASS stage or the recommendation for initial revascularization method. For bypass surgery in CLTI, appropriate arterial runoff to the lower limb and foot for wound perfusion and good-quality GSV for optimal autologous conduit are necessary conditions (Kobayashi et al. 2021). Unfortunately, our case did not satisfy either of these conditions.

Advancements in EVT include improved wires, sophisticated catheters, and multi-site retrograde access to allow therapy for complex disease patterns down to the foot (Machin et al. 2022). Retrograde access by distal puncture with either fluoroscopic or ultrasound guidance can certainly increase the ability to cross chronic total occlusions in IP arterial diseases. Moreover, the pedal artery angioplasty has been described as a salvage procedure for patients with CLTI in the presence of IM disease (Nakama et al. 2017a, b). Although hemodynamic stability remains the primary limitation of EVT for highly complex IP lesions, some CLTI patients can benefit from these procedures.

Our patient had severe gangrene in all the digits of his right foot, and TMA was an inevitable option from the beginning (Fig. 4e). Although our case showed IP grade 4 with multiple target vessels, the current version of GLASS does not consider multivessel IP revascularization due to lack of the evidence. Therefore, it is controversial how far we should revascularize in these types of IP diseases. We recognized the importance of pedal artery angioplasty for wound healing and believed that as much revascularization, if possible, would be beneficial for wound healing after TMA, and completed all the procedures as described above.

Multiple distal puncture sites have been developed for vascular access to-date, and their safety and efficacy have been demonstrated in clinical settings (Palena and Manzi, 2012, Nakama et al. 2017a, b; Schmidt et al. 2019). To be noted, distal puncture is not a method only for retrograde access. We can utilize the distal puncture site for both, entry and exit of a guidewire as shown in Fig. 3a and b (Needle Rendezvous technique) (Haraguchi et al. 2021). Moreover, by making pull-through formation, we can establish stronger back-up support for device delivery. We can also take advantage of BADFORM technique. In general, techniques associated with distal puncture are indispensable for successful EVT procedures especially in IP lesions.

At the same time, we also should consider some of the disadvantages of this method. One of them is a potential risk of causing vessel injury. Therefore, we should avoid applying this maneuver if there is only one artery remaining. Additionally, it is important to ensure the hemostasis management at the distal puncture sites. Some of them such as peroneal or metatarsal artery cannot be directly compressed from the body surface due to their anatomies. In those cases, we must successfully complete revascularization and balloon tamponade inside the vessel which is mandatory for hemostasis. We usually conduct balloon inflation for hemostasis for 10 min at the puncture sites of IM area concurrently performing vessel dilation. The shorter time may be sufficient for more distal puncture point such as dorsal metatarsal artery.

Thus, we have described a CLTI case with severely calcified IP disease in which successful revascularization was achieved by IM thrice distal puncture in a single EVT session. Individual techniques such as multi-site distal puncture in IM area, needle rendezvous technique, and BADFORM technique utilized in this case are previously reported, and are widely prevalent in daily clinical settings of EVT for IP lesions. However, the strategies adapted to pursue revascularization for severely calcified obstruction in the IP area is variable among the interventionalists all over the world. Through our case, we have demonstrated the possibility of using different EVT techniques and strategies for limb salvage.

## Conclusions

The success rate of revascularization can be substantially increased by effectively utilizing the retrograde approach in EVT for complex chronic total occlusions in IP arterial diseases.

## Data Availability

Not applicable.

## Abbreviations

ATA: Anterior tibial artery
BADFORM: Balloon deployment using forcible manner
CLTI: Critical limb threatening ischemia
DA: Dorsal artery
EVT: Endovascular treatment
GLASS: Global limb anatomic staging system
GSV: Great saphenous vein
GVG: Global vascular guideline
IM: Inframalleolar
IP: Infrapopliteal
PTA: Posterior tibial artery
SPP: Skin perfusion pressure
TMA: Transmetatarsal amputation
WIfI: Wound, Ischemia and foot Infection
